# Resistance to Plant-Parasitic Nematodes in Chickpea: Current Status and Future Perspectives

**DOI:** 10.3389/fpls.2019.00966

**Published:** 2019-07-24

**Authors:** Rebecca S. Zwart, Mahendar Thudi, Sonal Channale, Praveen K. Manchikatla, Rajeev K. Varshney, John P. Thompson

**Affiliations:** ^1^Centre for Crop Health, Institute for Life Sciences and the Environment, University of Southern Queensland, Toowoomba, QLD, Australia; ^2^Center of Excellence in Genomics and Systems Biology, International Crops Research Institute for the Semi-Arid Tropics, Hyderabad, India; ^3^Department of Genetics, Osmania University, Hyderabad, India

**Keywords:** *Cicer arietinum*, crop wild relatives, root-knot nematodes, cyst nematodes, root-lesion nematodes

## Abstract

Plant-parasitic nematodes constrain chickpea (*Cicer arietinum*) production, with annual yield losses estimated to be 14% of total global production. Nematode species causing significant economic damage in chickpea include root-knot nematodes (*Meloidogyne artiella*, *M. incognita*, and *M. javanica*), cyst nematode (*Heterodera ciceri)*, and root-lesion nematode (*Pratylenchus thornei*). Reduced functionality of roots from nematode infestation leads to water stress and nutrient deficiency, which in turn lead to poor plant growth and reduced yield. Integration of resistant crops with appropriate agronomic practices is recognized as the safest and most practical, economic and effective control strategy for plant-parasitic nematodes. However, breeding for resistance to plant-parasitic nematodes has numerous challenges that originate from the narrow genetic diversity of the *C. arietinum* cultigen. While levels of resistance to *M. artiella, H. ciceri*, and *P. thornei* have been identified in wild *Cicer* species that are superior to resistance levels in the *C. arietinum* cultigen, barriers to interspecific hybridization restrict the use of these crop wild relatives, as sources of nematode resistance. Wild *Cicer* species of the primary genepool, *C. reticulatum* and *C. echinospermum*, are the only species that have been used to introgress resistance genes into the *C. arietinum* cultigen. The availability of genomic resources, including genome sequence and re-sequence information, the chickpea reference set and mini-core collections, and new wild *Cicer* collections, provide unprecedented opportunities for chickpea improvement. This review surveys progress in the identification of novel genetic sources of nematode resistance in international germplasm collections and recommends genome-assisted breeding strategies to accelerate introgression of nematode resistance into elite chickpea cultivars.

## Introduction

Chickpea (*Cicer arietinum* L.) is a nutritionally rich cool-season pulse crop that plays an important role in ensuring global food security, as it is an important source of dietary protein. Chickpea also plays an important role in farming systems by fixing atmospheric nitrogen, contributing to soil fertility, acting as a disease break and controlling weeds. Currently, chickpea is grown in an area of over 14.5 Mha in 55 countries with total annual production of 14.7 Mt (FAO, 2017). India is the world’s largest consumer of chickpea and also the world’s largest producer, contributing over 70% of total global chickpea production (FAO, 2017). There are two types of chickpea differentiated by seed type and flower color, namely, desi and kabuli. Desi chickpeas have smaller dark colored seeds and pink flowers, and are predominantly grown in central Asia and in the Indian subcontinent. Whereas, kabuli chickpeas have larger beige seeds and white flowers and are predominantly grown in the Mediterranean region (Gaur et al., 2012). In India, chickpea is grown on residual moisture with low input management by resource-poor farmers (Singh and Reddy, 1991). The world average chickpea yield is less than 1 t/ha which is far less than the potential yield of 6 t/ha under favorable and irrigated conditions (Varshney et al., 2017). This enormous disparity between the actual and expected yield of chickpea is due to biotic stresses, caused by insects, bacteria, fungi, nematodes and viruses, and abiotic stresses, such as drought, nutrient deficiencies, salinity and chilling (Roorkiwal et al., 2016).

Globally, the loss of chickpea productivity due to plant parasitic nematodes is estimated to be 14% (Sasser and Freckman, 1987). Important elements for effective integrated control of plant-parasitic nematodes in cropping systems include (a) correct diagnosis of the nematode species, (b) effective rotations with non-hosts or fallow periods, and (c) use of tolerant and resistant crop cultivars (Thompson et al., 2000). Accurate diagnosis of nematode species requires extensive knowledge of nematode taxonomy and/or application of molecular diagnostic tools. Options for crop rotations are restricted in fields which are infested with nematode species with wide host ranges (Greco, 1987). Application of nematicides is avoided due to environmental and economic reasons. The most effective and sustainable long-term strategy to overcome constraints to chickpea production caused by plant-parasitic nematodes is the use of resistant cultivars. Resistance is the ability of a plant to reduce nematode reproduction such that, no nematode reproduction occurs in a highly resistant plant, a low level of reproduction occurs in a moderately resistant plant and unhindered nematode reproduction occurs in a susceptible plant (Roberts, 2002). Tolerance is a separately measured trait that characterizes the ability of a plant to grow and yield well even when infested with nematodes (Trudgill, 1991). Growing resistant cultivars has the advantage of preventing nematode reproduction and reducing yield losses in the current crop. Moreover, after growing resistant cultivars, nematode populations residual in the soil to damage subsequent crops are less than after susceptible cultivars, thus benefiting the whole farming system.

Advances in chickpea genomic resources resulting from the advent of next generation sequencing (NGS) technology, has the potential to greatly assist molecular breeding approaches to improve resistance to plant-parasitic nematodes and thereby help in achieving the yield potential of chickpea (Thudi et al., 2012). Recent reviews highlight the application of gene-editing technologies to control plant-parasitic nematodes (Leonetti et al., 2018) and improvements in chickpea genetic transformation technologies (Amer et al., 2019). In this review, we provide an overview of studies on the identification of nematode resistance genes in the *C. arietinum* cultigen and related species, focusing on three types of nematodes causing major economic damage to chickpea crops globally, namely, root-knot nematodes (*Meloidogyne artiella, M. incognita*, and *M. javanica*), chickpea cyst nematode (*Heterodera ciceri*) and root-lesion nematode (*Pratylenchus thornei*). We highlight the current status of nematode resistance in chickpea and discuss genomic tools available to improve the level of nematode resistance using genomic-assisted breeding.

## Chickpea-Nematode Interactions

Chickpea is a host for over 100 species of plant-parasitic nematodes (Nene et al., 1996; Sikora et al., 2018). However, only a small number of predominant species are considered to cause economic damage to chickpea crops throughout the world (Table 1). Crop damage due to nematode infestation can be challenging to diagnose because of non-specific above-ground plant symptoms seen on the plants (Sharma et al., 1992). The reduced functionality of the host plant roots due to the damage caused by plant-parasitic nematodes feeding and/or reproducing inside the root cells, results in infected plants showing the same symptoms as nutrient deficiency and water stress, namely, stunting, wilting, chlorotic leaves, reduced number of flowers and pods, reduced yield and patchiness in the field (Castillo et al., 2008). The significant root damage caused by plant-parasitic nematodes also reduces the ability of plants to cope with abiotic stresses of drought and low levels of plant nutrients in the soil.

**TABLE 1:** Geographic distribution of plant-parasitic nematodes infecting chickpea crops.

**Region/Country**		**Plant-parasitic nematodes**			
	Annual chickpea production (kt)^*^	**Root-knot nematodes (*Meloidogyne* spp.)**	**Cyst nematodes (*Heterodera* spp.)**	**Root-lesion nematodes (*Pratylenchus* spp.)**	**References**
**Mediterranean region**					
Turkey	455	*M. artiella*	*H. ciceri*	*P. thornei, P. mediterraneus, P. penetrans, P. zeae, P. brachyurus, P. alleni, P. alkan, P. erzurumensis*	Di Vito et al., 1994b
Syria	52	*M. artiella, M. arenaria*	*H. ciceri, H. rosii*	*P. thornei, P. mediterraneus*	Greco et al., 1988, 1992b
Italy	22	*M. artiella*		*P. thornei*	Greco, 1984
Spain	27	*M. artiella*	*H. goettingiana*	*P. thornei, P. penetrans, P. neglectus, P. minyus* (syn. *neglectus*)	Greco, 1984; Castillo et al., 1996; Nene et al., 1996
Jordan	2		*H. ciceri*		Di Vito et al., 2001
Lebanon	3		*H. ciceri*	*Pratylenchus*	Di Vito et al., 2001
**North Africa**					
Morocco	44	*M. artiella*		*P. thornei, P. mediterraneus, P. penetrans, P. zeae, P. ritteri*	Di Vito et al., 1994a
Algeria	20	*M. artiella*		*P. thornei, P. mediterraneus, P. penetrans, P. neglectus*	Di Vito et al., 1994a; Nene et al., 1996
Tunisia	5	*M. artiella*	*H. goettingiana*	*P. thornei, P. mediterraneus, P. penetrans*,	Di Vito et al., 1994a
Egypt	1	*M. artiella, M. incognita; M. javanica*			Nene et al., 1996
**East Africa**					
Ethiopia	444	*M. incognitia, M. javanica*			Sharma et al., 1992
Zimbabwe	0	*M. javanica*			Sharma et al., 1992
West Africa					
Malawi	67	*M. javanica*			Sharma et al., 1992
**South Asia**					
India	7,819	*M. incognita, M. javanica, M. arenaria*	*H. swarupi, H. cajani*	*P. thornei, P. mulchandi, P. coffeae, P. zeae*	Sharma and McDonald, 1990; Ali, 1995; Castillo et al., 2008
Nepal	11	*M. incognita, M. javanica*			
Pakistan	517	*M. incognita, M. javanica*			
Bangladesh	8	*M. incognita, M. javanica*			
Myanmar				*P. thornei*	Nene et al., 1996
**Australasia**					
Australia	875			*P. thornei, P. neglectus, P. brachyurus*	Nene et al., 1996
**North America**					
United States	108		*H. goettingiana*	*P. neglectus*,	Nene et al., 1996
Mexico	122			*P. thornei*	
**South America**					
Brazil	–	*M. incognita, M. javanica*		*P. brachyurus*	Sharma and McDonald, 1990; Nene et al., 1996

^*^Source: (FAO, 2017).

Plant-parasitic nematodes contribute to decreased plant vigor by reducing *Rhizobium* root nodulation and nitrogen-fixing ability of the host plant (Tiyagi and Parveen, 1992; Vovlas et al., 1998; Wood et al., 2018). Furthermore, plant-parasitic nematodes exacerbate crop damage caused by other biotic stresses. Nematode infection leads to enhanced severity of infection with soil-borne fungal pathogens causing Fusarium wilt (*Fusarium oxysporum* f. sp. *ciceris*) (Castillo et al., 1998, 2003) and dry root rot (*Rhizoctonia bataticola*) (Ali and Sharma, 2003).

### Root-Knot Nematodes

**FIGURE 1:**
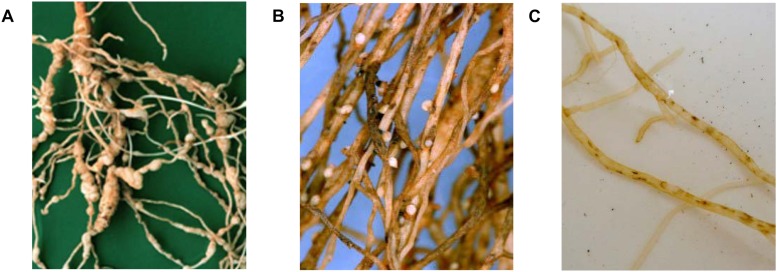
Visual symptoms of nematode infection in chickpea roots. **(A)** Galled roots caused by *Meloidogyne incognita* (source: *P. Castillo*). **(B)** Cysts caused by *Heterodera ciceri* (source: ICARDA). **(C)** Necrotic lesions caused by *Pratylenchus thornei* (source: DPIRD).

Root-knot nematodes, *Meloidogyne* spp., rank as the most economically damaging nematodes to agricultural crops worldwide due to their broad host range and wide geographical distributions (Jones et al., 2013). Root-knot nematodes are sedentary endoparasites. Many *Meloidogyne* species are parthenogenic or facultatively parthenogenic. Motile male and female second stage juveniles penetrate the root surface. Female root-knot nematodes migrate to the vascular tissue and establish permanent feeding sites called giant cells (Vovlas et al., 2005). As the juveniles feed they become swollen and at maturity they produce egg masses that contain up to 600 eggs (Hernández Fernández et al., 2005). The characteristic galls on infected roots (Figure 1A) contain four to six giant cells that are formed by repeated nuclear division without cell division. Galls induced by *M. artiellia* on chickpea roots are smaller than those produced by other root-knot species (Vovlas et al., 2005).

*Meloidogyne incognita* and *M. javanica* are the most prevalent species of root-knot nematodes in tropical chickpea growing countries, including Ethiopia, Zimbabwe and Malawi in Africa (Sharma et al., 1992), India, Nepal, Pakistan and Bangladesh in South Asia (Castillo et al., 2008) and Brazil in South America (Sharma and McDonald, 1990; Table 1). In India, *M. arenaria* also causes severe damage to chickpea crops (Castillo et al., 2008). *M. artiellia* is the most widespread root-knot nematode species in cooler chickpea growing countries of the Mediterranean region, including Italy, Spain, Syria, Turkey, Morocco, Algeria, and Tunsia (Greco et al., 1992b; Di Vito et al., 1994a, b). The root-knot nematodes, *M. incognita* and *M. javanica*, cause yield losses of 19 to 40% to chickpea in India (Ali and Sharma, 2003) with thresholds for damage for these species varying from 200 to 2000 eggs and/or juveniles per liter soil at the time of sowing (Sharma et al., 1992). On the other hand, the damage threshold for *M. artiellia* is calculated to be considerably lower at 20 to 140 eggs and juveniles per liter of soil, with 2000 nematodes per liter at planting resulting in yield losses of 50 to 80% (Di Vito and Greco, 1988).

### Cyst Nematodes

Chickpea cyst nematode, *H. ciceri*, is the most damaging cyst nematode infecting chickpea, although several other *Heterodera* spp. have been reported on or in the rhizosphere of chickpea without causing damage (Table 1), namely, *H. cajani* and *H. swarupi* in India (Ali and Sharma, 2003) and *H. goettingiana* in Tunisia and Morocco (Di Vito et al., 1994a). Cyst nematodes are sedentary semi-endoparasites. Motile juvenile nematodes penetrate the root surface and move to the vascular tissue where they form a permanent feeding site characterized by syncytia cells (Greco et al., 1992a). Swollen females rupture root tissues with the posterior portion of their bodies, which then protrude from the root surface forming visual cysts about 0.5 to 1.0 mm in diameter. The females retain eggs inside their bodies. While only one generation is completed per growing season on chickpea, each cyst contains up to 300 eggs (Kaloshian et al., 1986). Moreover, eggs can survive long periods in the soil in the absence of a host (Castillo et al., 2008). Infected chickpea roots are characterized by the visible swollen adult females protruding from the root surface (Figure 1B). The lemon shaped cysts change from white to brown as females mature (Kaloshian et al., 1986).

*Heterodera ciceri* is distributed throughout the eastern Mediterranean region in Turkey (Di Vito et al., 1994b), Syria (Greco et al., 1992b), Jordan and Lebanon (Di Vito et al., 2001). While *H. ciceri* predominantly affects chickpea (Greco et al., 1986), other grain legumes, fodder species and ornamental plants have been reported as hosts (Di Vito et al., 2001). *H. ciceri* was the most damaging plant-parasitic nematode in chickpea crops in Syria (Greco et al., 1992b). *H. ciceri* is aggressive on chickpea crops with economic yield losses occurring with 1000 eggs per liter soil. Moreover, yield losses of 20, 50, 80, and 100% were reported to occur with 8000, 16000, 32000, and 64000 eggs per liter soil at planting, respectively (Greco et al., 1988).

### Root-Lesion Nematodes

Root-lesion nematodes are the predominant plant-parasitic nematode found in chickpea crops in surveys in North Africa (Di Vito et al., 1994a), Turkey (Di Vito et al., 1994b), and Spain (Castillo et al., 1996). Root-lesion nematodes are migratory endoparasites that cause extensive damage to cortical cells in the pathway of migration and during feeding (Castillo et al., 1998). In the species *P. thornei* male nematodes are rare and females reproduce by mitotic parthenogenesis, depositing eggs in the cavities of root cells caused by nematode feeding and movement. *P. thornei* takes 25 to 35 days to complete its life cycle at 20 to 25°C on carrot disk culture (Castillo et al., 1995); thus several generations can occur in a growing season (Sikora et al., 2018). *P. thornei* eggs and nematodes can survive in the soil in the absence of host plants. If the soil dries slowly a high proportion of the nematodes can survive the dry conditions (Thompson et al., 2017, 2018). Infection by *P. thornei* is characterized by dark brown to black lesions on chickpea roots (Figure 1C). Damage caused by root-lesion nematodes is generally less obvious than that caused by root-knot or cyst nematodes (Sharma et al., 1992) and symptoms of *P. thornei* damage to the roots do not always result in visible symptoms on above-ground plant parts. The wide host range of root-lesion nematodes hampers management strategies.

*Pratylenchus thornei* is the predominant species of root-lesion nematode causing damage to chickpea crops throughout the world. The distribution of *P. thornei* extends throughout major chickpea growing countries, including Australia (Thompson et al., 2000), India (Sharma et al., 1992), North Africa (Di Vito et al., 1994a), Turkey (Di Vito et al., 1994b), and Spain (Castillo et al., 1996). In India, the world’s largest producer and consumer of chickpea, *P. thornei* is emerging as a serious threat to chickpea production, with high populations reported in Madhya Pradesh (Baghel and Singh, 2013), Rajasthan (Ali and Sharma, 2003), Maharashtra (Varaprasad et al., 1997), and Uttar Pradesh (Sebastian and Gupta, 1995). Numerous other *Pratylenchus* species have been reported in surveys of chickpea crops in North Africa and the Mediterranean region, Brazil and North America (Table 1), however, limited information is available on the extent of crop damage they cause. The species *P. thornei* infects many cereal and pulse crops (Sikora et al., 2018); thus high populations can build up quickly in the soil and affect the whole farming system. In Australia, where *P. thornei* is ranked as the second most economically important biotic stress affecting chickpea (Murray and Brennan, 2012), yield losses of 25% were obtained in chickpea fields with 11600 *P. thornei*/kg of soil at planting (Thompson et al., 2000; Reen et al., 2014). A damage threshold as low as 31 nematode per liter of soil was reported for *P. thornei* by Di Vito et al. (1992) in field conditions in Syria, with 2000 nematodes per liter at planting resulting in yield losses up to 58%.

## Sources of Nematode Resistance

Accurate, reliable phenotyping is essential for screening germplasm to identify sources of resistance. Accurate phenotyping experiments require robust statistical design in a controlled environment with plants inoculated with a known initial population of nematodes and/or eggs. Resistance to root-knot nematode is generally quantified by visual inspection and rating of infected roots using a root-galling index on a 1 to 5 scale (with 1 = no galls and 5 = greater than 100 galls per root) (Rao and Krishnappa, 1995; Hassan and Devi, 2004; Haseeb et al., 2006; Chakraborty et al., 2016). In addition to scoring root-galling index, Sharma et al. (1992, 1993, 1995) evaluated gall size (on a 1–9 scale with 1 = no galls and 9 = very large galls) and percent galled area (on a 1 to 9 scale with 1 = no galls and 9 = more than 50% root area galled) to calculate a root damage index, as an average of the three ratings. Mechanisms of resistance, such as increased peroxidase activity of infected roots, have also been used to screen chickpea germplasm against root-knot nematode (Siddiqui and Husain, 1992; Chakrabarti and Mishra, 2002). The resistance level of a plant to chickpea cyst nematode is determined by rating the number of females and cysts on infected roots using a 0 to 5 scale (with 0 = no females and cysts and 5 = greater than 50 females and cysts) (Di Vito et al., 1988; Singh et al., 1989). In the case of migratory root-lesion nematodes, the nematodes need to be extracted from roots and/or soil before quantification is possible. Researchers have reported resistance levels to *P. thornei* in relation to reproduction factor (final nematode population/initial nematode population) (Tiwari et al., 1992; Di Vito et al., 1995), or as number of nematodes per unit of root and/or soil (Thompson et al., 2011; Reen et al., 2019). Measuring visual lesions present on infected roots (Ali and Ahmad, 2000), is not recommended as lesions are only symptoms and not a direct measure of nematode numbers.

### *Cicer arietinum* Cultigen

To date, there has been relatively little success in identifying resistance to plant-parasitic nematodes in the *C. arietinum* cultigen, namely, chickpea cultivars, breeding lines and landraces held in global genebanks, compared with the number of accessions that have been evaluated (Table 2). Extensive screening efforts in Syria by the International Center for Agricultural Research in the Dry Areas (ICARDA) and the Institute for Sustainable Plant Protection, Italy, have been devoted to identifying resistance to *H. ciceri*, the most devastating nematode to chickpea production in the Mediterranean region. Despite screening close to 10000 chickpea accessions from global germplasm collections held by ICARDA and the International Crop Research Institute for the Semi-Arid Tropics (ICRISAT), none were found to be resistant (Di Vito et al., 1996; Singh et al., 1996) and merely 20 lines were rated as moderately resistant to *H. ciceri* (Di Vito et al., 1988).

**TABLE 2:** Studies to identify resistance to root-knot nematodes (*Meloidogyne incognita*, *M. javanica*), cyst nematode (*Heterodera ciceri*), and root-lesion nematode (*Pratylenchus thornei*) in the *Cicer arietinum* cultigen.

**Species**	**Total no. of lines screened**	**No. of lines**		**Source of germplasm**	**References**
		**Resistant**	**Moderately resistant**		
*M. incognita*	20	0	0	Indian cultivars	Siddiqui and Husain, 1992
	13	0	0	Indian cultivars	Rao and Krishnappa, 1995
	108	0	0	Indian Agricultural Research Institute, Delhi; Indian Institute of Pulse Research, Kanpur; Rajasthan College of Agriculture; Mohanlal Sukhadia University, Udaipur; Mahatma Phule Krishi Vidyapeeth, Rahuri, India	Mhase et al., 1999
	10	0	0	Indian cultivars	Chakrabarti and Mishra, 2002
	72	58	0	Indian Institute of Pulse Research, Kanpur, India	Hassan and Devi, 2004
	32	6	32	Indian Institute of Pulse Research, Kanpur, India	Haseeb et al., 2006
	34	17	60	Indian Institute of Pulse Research, Kanpur, India	Chakraborty et al., 2016
*M. javanica*	1,000	0	0	ICRISAT, India	Sharma et al., 1992
	178	0	0	ICRISAT, India	Sharma et al., 1993
	47	0	0	ICRISAT, India	Sharma et al., 1995
	600	0	0	ICRISAT and Indian Institute of Pulse Research, India	Ali and Ahmad, 2000
	10	0	0	ICRISAT, India	Bhagwat and Sharma, 2001
	10	0	0	National Agricultural Research Council, Pakistan	Hussain et al., 2001
	7,000	0	0	ICRISAT, India	Ansari et al., 2004
*H. ciceri*	2,001	0	20	ICARDA, Syria	Di Vito et al., 1988
	7,258	0	0	ICARDA, Syria	Di Vito et al., 1996
*P. thornei*	215	35	68	Indian Institute of Pulse Research, Kanpur; JNKVV Jabalpur, India	Tiwari et al., 1992
	600	0	17	ICRISAT and Indian Institute of Pulse Research, India	Ali and Ahmad, 2000
	453	1	14	ICARDA; ICRISAT; Australian cultivars and breeding lines	Thompson et al., 2011

Screening efforts focusing on identifying resistance to *M. javanica* in the *C. arietinum* germplasm collection held in the ICRISAT genebank proved futile, with no resistance identified in numerous studies testing several thousand accessions (Sharma et al., 1992, 1993, 1995; Ali and Ahmad, 2000; Bhagwat and Sharma, 2001; Ansari et al., 2004). Nonetheless, a few susceptible lines were deemed tolerant to *M. javanica* and produced a higher yield and shoot biomass in *M. javanica*-infested soil, even though the roots supported nematode reproduction (Sharma et al., 1992, 1993, 1995). Hussain et al. (2001) screened ten chickpea cultivars from Pakistan for resistance to *M. javanica*, and found all ten cultivars showed a moderate level of resistance.

Early studies were unsuccessful in finding resistance to *M. incognita* in Indian chickpea cultivars (Siddiqui and Husain, 1992; Rao and Krishnappa, 1995; Mhase et al., 1999; Chakrabarti and Mishra, 2002). However, more recent studies have reported resistance and moderate resistance to *M. incognita* in Indian chickpea cultivars and breeding lines (Hassan and Devi, 2004; Haseeb et al., 2006; Chakraborty et al., 2016). Considering the broad host range and widespread occurrence of this nematode species in India (Khan et al., 2014) it is plausible that incidental selection for resistance to *M. incognita* has occurred in more recent breeding programs. Sikora et al. (2018) reported that no attempts have been made to screen chickpea germplasm for resistance to *M. artiella*.

Sources of resistance and moderate resistance to *P. thornei* in the *C. arietinum* cultigen have been identified in breeding lines in India (Tiwari et al., 1992; Ali and Ahmad, 2000) and in accessions in the ICRISAT genebank in India (Ali and Ahmad, 2000) and Australia (Thompson et al., 2011).

The limited diversity of resistance genes in the *C. arietinum* cultigen is not restricted to plant-parasitic nematodes. *C. arietinum* lacks diversity for a range of biotic and abiotic stresses (Smýkal et al., 2015). Abbo et al. (2003) proposed that this low level of diversity can be attributed to the following genetic bottlenecks that occurred during the evolution and domestication of chickpea: (i) there is a limited distribution of chickpea wild progenitor species, (ii) the founder effect arising from the domestication of only a small number of wild genotypes, which is a bottleneck common to all modern crops, (iii) a shift from winter to spring phenology to avoid devastation by Ascochyta blight (*Ascochyta rabiei*), and (iv) the substitution of a large number of landraces with a small number of elite cultivars from modern breeding caused yet further reduction in the diversity of the *C. arietinum* genepool.

The availability of large and diverse germplasm collections is a key element for the successful identification of disease resistant lines (Infantino et al., 2006). Landraces, traditional locally adapted varieties that lack formal crop improvement (Villa et al., 2005), serve as a valuable genetic resource that may help widen the narrow genetic base of chickpea by circumventing the genetic bottlenecks caused by changing from winter to spring phenology and modern breeding. While landraces hold much genetic diversity of the *C. arietinum* cultigen, strategic methods are crucial to mine the global chickpea germplasm collections, which have conserved close to a hundred thousand accessions (Smýkal et al., 2015). Recent developments of core, reference and mini-core collections (Upadhyaya et al., 2001, 2008) and subsampling strategies such as the focused identification of germplasm strategy (FIGS) (Khazaei et al., 2013) have created unprecedented opportunities for the systematic screening of a practical number of accessions.

A core collection is defined as a subset of all the accessions representing the genetic diversity of crop species and wild relatives with minimum repetition (Frankel and Brown, 1984). It constitutes about 10% of the total number of accessions and represents genetic diversity of the entire global germplasm collection. Based on geographic distribution and quantitative traits of accessions held at ICRISAT, a core subset was developed consisting of 1956 accessions of chickpea (Upadhyaya and Ortiz, 2001). However, the size of the core collection was still too large to be systematically evaluated for traits of interest. To overcome this limitation, a mini-core collection was developed where a subset of 211 accessions (1.1% of the entire collection) was selected based on taxonomic, morphological and geographic data (Upadhyaya and Ortiz, 2001). Also, a composite collection of 3000 accessions was formed, which represents the diversity of accessions held at ICRISAT and ICARDA collectively. From this collection, the ‘Reference Set,’ was produced, composed of the full mini-core collection (211) and an additional 82 *C. arietinum* accessions, plus four *C. reticulatum* and three *C. echinospermum* genotypes (Upadhyaya et al., 2006).

The chickpea mini-core collection and Reference Set have been phenotyped in several studies to identify traits of interest to combat biotic and abiotic stresses. These traits include resistance to multiple diseases of economic concern namely, Ascochyta blight, Fusarium wilt, dry root rot and Botrytis gray mold (Pande et al., 2006), as well as root architectural traits for optimal use of soil resources, and adaptation to drought and other abiotic challenges (Kashiwagi et al., 2005; Krishnamurthy et al., 2010, 2011). In addition to identifying germplasm with traits of interest, these collections have been used to understand the genetic basis of heat and drought tolerance traits by using genome-wide association studies (GWAS) and candidate gene-based mapping approaches (Thudi et al., 2014). These valuable repositories of germplasm covering the genetic diversity of *C. arietinum* offer opportunities to efficiently search for sources of resistance to plant-parasitic nematodes that were not previously available.

### Wild *Cicer* Relatives

Chickpea wild relatives can be used to reintroduce traits and widen the genetic base of the *C. arietinum* cultigen that did not pass through the domestication bottleneck (Abbo et al., 2003). The genus *Cicer* comprises 44 species, of which nine are annuals and 35 perennials (Smýkal et al., 2015). Annual *Cicer* species in the primary genepool *(C. arietinum, C. reticulatum*, and *C. echinosperum*) are cross-compatible, while those in the secondary genepool (*C. bijugum, C. pinnatifidum*, and *C. judaicum*) and tertiary genepool (*C. chorassanicum, C. cuneatum*, and *C. yamashitae*) have barriers to hybridization with *C. arietinum* (Croser et al., 2003). Despite this, accessions from all three genepools held in germplasm collections have been screened for resistance to plant-parasitic nematodes (Table 3).

**TABLE 3:** Studies to identify resistance to root-knot nematodes (*Meloidogyne artiellia*, *M. javanica*), cyst nematode (*Heterodera ciceri*), and root-lesion nematode (*Pratylenchus thornei*) in *Cicer* wild relatives.

**Nematode species**	**Genepool**	***Cicer* species**	**Total no. of lines screened**	**No. of lines**		**References**
				**Resistant**	**Moderately resistant**	
*M. artiellia*	Primary	*C. echinospermum*	1	0	0	
		*C. reticulatum*	15	0	0	
	Secondary	*C. bijugum*	32	1	5	Di Vito et al., 1995
		*C. judaicum*	31	0	0	
		*C. pinnatifidum*	23	1	3	
		*C. chorassanicum*	3	0	3	
	Tertiary	*C. cuneatum*	3	0	1	
		*C. yamashitae*	3	0	0	
*M. javanica*	Primary	*C. reticulatum*	3	0	0	Sharma et al., 1993
	Secondary	*C. bijugum*	2	0	0	
		*C. judaicum*	14	0	0	
		*C. pinnatifidum*	4	0	0	
	Tertiary	*C. chorassanicum*	1	0	0	
		*C. cuneatum*	1	0	0	
*H. ciceri*	Primary	*C. echinospermum*	1	0	0	Di Vito et al., 1988
		*C. reticulatum*	2	0	0	
	Secondary	*C. bijugum*	3	0	2	
		*C. judaicum*	6	0	0	
		*C. pinnatifidum*	5	0	0	
		*C. chorassanicum*	1	0	0	
	Tertiary	*C. cuneatum*	1	0	0	
		*C. yamashitae*	1	0	0	
	Primary	*C. echinospermum*	4	0	0	Singh et al., 1989
		*C. reticulatum*	23	0	0	
	Secondary	*C. bijugum*	23	21	0	
		*C. judaicum*	47	0	0	
		*C. pinnatifidum*	30	0	0	
		*C. chorassanicum*	5	0	0	
	Tertiary	*C. cuneatum*	3	0	0	
		*C. yamashitae*	2	0	0	
	Primary	*C. echinospermum*	8	0	0	Di Vito et al., 1996
		*C. reticulatum*	36	1	0	
	Secondary	*C. bijugum*	13	1	0	
		*C. judaicum*	18	0	0	
		*C. pinnatifidum*	18	6	0	
		*C. chorassanicum*	3	0	0	
	Tertiary	*C. cuneatum*	3	0	0	
		*C. yamashitae*	3	0	0	
*P. thornei*	Primary	*C. echinospermum*	1	0	0	Di Vito et al., 1995
		*C. reticulatum*	34	0	0	
	Secondary	*C. bijugum*	32	6	7	
		*C. judaicum*	38	11	9	
		*C. pinnatifidum*	31	0	0	
		*C. chorassanicum*	5	0	1	
	Tertiary	*C. cuneatum*	3	3	0	
		*C. yamashitae*	3	1	1	
	Primary	*C. echinospermum*	15	0	2	Thompson et al., 2011
		*C. reticulatum*	52	0	2	
	Secondary	*C. bijugum*	35	0	6	
		*C. pinnatifidum*	1	0	0	
	Primary	*C. echinospermum*	41	3	11	Reen et al., 2019
		*C. reticulatum*	133	10	29	

In search for resistance to *H. ciceri*, a limited number of wild *Cicer* relatives were screened. Singh et al. (1989) screened accessions from all 8 annual wild *Cicer* species and identified a high level of resistance to *H. ciceri* only in accessions of *C. bijugum*. However, screening of additional germplasm identified resistance to *H. ciceri* in one accession of *C. reticulatum*, one of *C. bijugum* and six of *C. pinnatifidum* (Di Vito et al., 1996). The resistance from the cross-compatible *C. reticulatum* accession was then successfully transferred to *C. arietinum* breeding lines (Singh et al., 1996; Malhotra et al., 2002, 2008). Di Vito et al. (1995) reported resistance to *P. thornei* in accessions from the secondary genepool (*C. bijugum* and *C. judaicum*) and tertiary genepool (*C. cuneatum* and *C. yamashitae*), while no resistance was found in accessions from the primary genepool (*C. echinosperum* and *C. reticulatum*). Thompson et al. (2011) identified moderate resistance to *P. thornei* in accessions from both *C. echinosperum* and *C. reticulatum* in the primary genepool, as well as accessions of *C. bijugum*. Successful hybridizations of these *C. echinosperum* and *C. reticulatum* accessions with *C. arietinum* in the Australian chickpea breeding program has produced breeding lines with resistance at a level equivalent to the *Cicer* wild relative parents (Thompson et al., 2011; Rodda et al., 2016). To date, no sources of resistance to root-knot nematodes have been identified in the *Cicer* primary genepool. Resistance to *M. artiellia* has been identified in one accession of *C. bijugum* and one accession of *C. pinnatifidum* from the ICARDA genebank (Di Vito et al., 1995). No resistance was found for *M. javanica* in wild *Cicer* relatives screened by Sharma et al. (1993).

Using embryo rescue and tissue culture techniques, hybrids between *C. arietinum* and accessions of secondary genepool species *C. bijugum*, *C. judaicum*, and *C. pinnatifidum* are possible (Ahmad and Slinkard, 2004; Clarke et al., 2006). However, these techniques are extremely inefficient. Many crosses are required to recover hybrids and the few hybrids that are recovered are affected by androgenesis, infertility and lack of vigor (Clarke et al., 2011). Thus, further advancements in techniques are required to increase efficiency and cross the barriers to hybridization that exist between accessions of the secondary genepool and the *C. arietinum* cultigen before these sources of resistance can be applied in chickpea breeding (Pratap et al., 2018). For now, the only accessible sources of wild *Cicer* germplasm are accessions of *C. echinosperum* and *C. reticulatum*. However, Berger et al. (2003) highlighted the limited number of unique accessions of these wild *Cicer* species held in international genebanks. Of 43 *C. echinosperum* accessions in the world collection, only 13 are original independent accessions, with the remainder being duplicates under different accession numbers used by different genebanks. Of 139 *C. reticulatum* accessions, only 18 were original accessions. This under-representation of wild *Cicer* relatives in global genebank collections has been recently addressed with new collecting expeditions for *C. echinosperum* and *C. reticulatum* in south-eastern Turkey spanning the geographic range of these wild *Cicer* species (von Wettberg et al., 2018). Reen et al. (2019) recently demonstrated the value of this collection for increasing genetic diversity for resistance to plant-parasitic nematodes. Thirteen accessions were identified as significantly more resistant to *P. thornei* (*P* < 0.05) than the previously most resistant *C. echinosperum* accession reported by Thompson et al. (2011). Moreover, wild introgression populations of *C. echinosperum* and *C. reticulatum* parents into *C. arietinum* using elite chickpea varieties adapted to the major chickpea growing regions of the world, namely, India, Australia, Turkey, Ethiopia, and Canada (von Wettberg et al., 2018), will be invaluable resources for the identification and utilization of traits of interest in wild *Cicer* relatives, including resistance to plant-parasitic nematodes.

## Chickpea Genomic Resources

### Molecular Marker-Based Resources

Recent advances in genomics research have enabled the development and application of molecular markers for crop improvement (Thudi et al., 2014; Varshney et al., 2018b). In the case of chickpea, *2n* = *2x* = 16 chromosomes and a genome size of ∼738 Mb (Varshney et al., 2013b), extensive genomic and transcriptomic resources have been developed (Varshney et al., 2009; Nayak et al., 2010; Hiremath et al., 2011; Thudi et al., 2011; Kudapa et al., 2014; Agarwal et al., 2016; Mashaki et al., 2018). The availability of these resources has facilitated the development of molecular markers and high density genetic maps in chickpea (Thudi et al., 2011; Varshney et al., 2014b; Jaganathan et al., 2015; Kale et al., 2015). Over 2000 simple sequence repeat (SSR) markers, millions of single nucleotide polymorphism (SNP) markers, and over 15000 diversity array technology (DArT) markers, have been developed for chickpea (Varshney, 2016) in the last decade. These molecular markers and genetic linkage maps, in combination with phenotypic data and quantitative trait loci (QTL) analysis, have been used to identify genomic regions responsible for complex traits in chickpea like drought tolerance (Varshney et al., 2014b), salinity tolerance (Vadez et al., 2012; Pushpavalli et al., 2015), heat tolerance (Paul et al., 2018), early flowering (Mallikarjuna et al., 2017), vernalization (Samineni et al., 2016) and resistance to Fusarium wilt and Ascochyta blight (Sabbavarapu et al., 2013). Further, using a GWAS approach, markers associated with drought and heat tolerance traits (Thudi et al., 2014) and protein content (Jadhav et al., 2015) have also been reported. Besides using molecular markers to assist understanding molecular mechanisms of different traits, several functional genomics approaches, such as suppression subtractive hybridization (SSH), super serial analysis of gene expression (SuperSAGE), microarray, and expressed sequence tags (EST) sequencing were also recently applied to chickpea (Buhariwalla et al., 2005; Molina et al., 2008; Varshney et al., 2009). These molecular marker-based resources, when coupled with robust and accurate phenotyping to detect marker-trait associations, can be applied to chickpea breeding to (i) assist the indirect selection of nematode resistance, (ii) facilitate pyramiding of resistance genes from several resistant or moderately resistant sources to provide cultivars with durable nematode resistance, and (iii) combine resistance to multiple biotic stresses.

### Next-Generation Sequencing-Based Resources

Several key traits have been targeted for transcriptomic studies in chickpea (Varshney et al., 2009; Hiremath et al., 2011; Kudapa et al., 2014; Kaashyap et al., 2018). In recent years, sequencing and *de novo* assembly of the chickpea transcriptome using short-reads and high-throughput small RNA sequencing were also deployed to discover tissue-specific and stress-responsive expression profiles (Jain et al., 2014; Kohli et al., 2014). These functional genomic resources were also used to develop informative SSR and SNP markers in chickpea (Agarwal et al., 2012; Hiremath et al., 2012; Jhanwar et al., 2012; Garg et al., 2014; Kudapa et al., 2014; Pradhan et al., 2014; Parida et al., 2015). Recently, a Gene Expression Atlas (CaGEA) from 27 chickpea tissues across five developmental stages, namely, germination, seedling, vegetative, reproductive, and senescence, of a chickpea breeding cultivar, ICC 4958, has been developed (Kudapa et al., 2018). Ramalingam et al. (2015) extensively reviewed several studies on application of proteomics and metabolomics in chickpea and other crop legumes. Integration of these technologies with genomics has the potential to inform the molecular mechanisms of plant responses to biotic stresses such as nematode infestation and identify key candidate genes to be introgressed for chickpea improvement.

Following the release of the draft genomes of chickpea (Jain et al., 2013; Varshney et al., 2013b), efforts have been made during the last decade to improve the genome assemblies. For instance, Ruperao et al. (2014) using sequence data from flow cytometry isolated chromosomes to identify misplaced contigs for improving and validating the desi and kabuli draft chickpea genome assemblies. Similarly, Parween et al. (2015), using additional sequence data and improved genetic maps, developed an improved version of the desi genome assembly. In addition, a draft genome assembly of *C. reticulatum* the wild progenitor of chickpea has been recently reported (Gupta et al., 2017). Further, in order to design new strategies to harness the existing genetic diversity in germplasm lines conserved in genebanks across the world, re-sequencing of germplasm lines has been advocated (McCouch et al., 2013). Toward this direction in chickpea, 90 elite lines, 35 parental genotypes of mapping populations, and 129 released varieties have been re-sequenced (Varshney et al., 2013b, 2019; Thudi et al., 2016a,b). Moreover, efforts are currently underway at ICRISAT to re-sequence the 3000 germplasm lines of the composite chickpea collection. Next-generation sequencing-based genomic resources can provide insights into candidate genes determining nematode resistance and in this way enable diagnostic markers for accurate and efficient indirect selection of resistance to be developed. Furthermore, insights into candidate resistance genes will enable mechanisms of resistance to plant-parasitic nematodes to be deciphered. Increased knowledge of the mechanisms of resistance in different germplasm sources would allow the possibility to breed for enhanced durability of nematode resistance by combining genes for different resistance mechanisms in the one chickpea cultivar.

### Genome-Assisted Breeding

Molecular breeding approaches utilizing markers and the large-scale genetic and genomic resources that are now available for chickpea have been successful in improving chickpea for target traits. Some superior lines with enhanced tolerance or resistance to abiotic and biotic stresses as well as agronomically important traits have been successfully developed in legumes using marker-assisted backcrossing (MABC) (Lucas et al., 2015; Varshney, 2016; Varshney et al., 2018a). A genomic region in chickpea (known as “*QTL-hotspot*”) harboring several QTL for drought component traits was identified (Varshney et al., 2014b) and successfully introgressed initially into JG 11, an elite Indian chickpea cultivar (Varshney et al., 2013a). Preliminary yield trials indicated a 12 to 24% increase in yield under drought conditions. In addition, the introgression of this genomic region into different genetic backgrounds, like chickpea cultivars KAK 2 and Chefe, was also found to enhance drought tolerance. Further, this genomic region is being introgressed into elite cultivars in Kenya, Ethiopia and India (Thudi et al., 2017). Molecular breeding lines with enhanced resistance to Fusarium wilt (Pratap et al., 2017; Mannur et al., 2019) and Ascochyta blight in different elite genetic backgrounds (Varshney et al., 2014a) have been developed. ICRISAT has also developed highly cost-effective 10 SNP panels for several traits in legumes including chickpea that can be used for early generation selection to accelerate the efficiency of selection in breeding programs, besides cost-effective high-throughput genotyping platforms (Roorkiwal et al., 2018). This 10 SNP panel is being used extensively in early generation selection in south Asia and Sub-Saharan Africa. Identification of molecular markers associated with nematode resistance will enable genomics-assisted breeding to facilitate the introgression of nematode resistance in elite chickpea cultivars in breeding programs worldwide.

## Future Perspectives

In this review we have outlined progress in the discovery of resistance to plant-parasitic nematodes in various germplasm sources suitable for introgression into chickpea cultivars. Screening a large number of germplasm lines is expensive and time-consuming. In the past this has either limited the number of lines that have been evaluated for nematode resistance or required large investments in resources and effort. The development of the chickpea mini-core and reference set germplasm collections of landraces and *C. arietinum* breeding lines, provides cost-effective and manageable entry points into the vast global chickpea germplasm collections (Gaur et al., 2012). Although major genetic bottlenecks may have contributed to the lack of genetic diversity for resistance against plant-parasitic nematodes available in the *C. arietinum* cultigen, new opportunities exist to widen the genetic base of chickpea for traits of interest. The small number of wild genotypes contributing to the domesticated *C. arietinum* cultigen can be circumvented by evaluating recent collections of chickpea wild species *C. reticulatum* and *C. echinospermum* for resistance to plant-parasitic nematodes.

To the best of our knowledge, no information is currently available on the nature of inheritance and genetics of plant-parasitic nematode resistance genes in chickpea. Considerable advancements in chickpea genomic resources since the majority of the past efforts to identify sources of resistance to various nematode species, provide unprecedented opportunities to accelerate identification and characterization of nematode resistance genes. Availability of an extensive number of molecular markers and genomic resources in chickpea, coupled with robust phenotyping, will facilitate identification of markers linked with resistance to plant-parasitic nematodes. Identification of candidate genes for nematode resistance could provide diagnostic markers that could be used for indirect selection of nematode resistance. Furthermore, genomic tools can provide insights into the mechanisms of resistance to plant-parasitic nematodes in chickpea. Identification of marker-trait associations will facilitate rapid introgression of resistance to plant-parasitic nematodes and adoption of genomics-assisted breeding into chickpea breeding programs world-wide. Sources of moderate resistance can be dissected with molecular markers to identify minor genes. If additive in gene action, sources of moderate resistance could be successfully combined using genomics-assisted selection to produce nematode resistant chickpea cultivars. We have indicated a number of successes in the identification of resistance to plant-parasitic nematodes that provide encouragement to apply and exploit genomic tools and intensify efforts to have resistant cultivars available to growers in all regions where plant-parasitic nematodes diminish production of chickpea and of other host crops grown in rotation.

## Author Contributions

All authors contributed to sections of the manuscript according to their expertise and have edited, read, and approved the submitted version.

## Conflict of Interest Statement

The authors declare that the research was conducted in the absence of any commercial or financial relationships that could be construed as a potential conflict of interest.
